# MetaHCR: a web-enabled metagenome data management system for hydrocarbon resources

**DOI:** 10.1093/database/bay087

**Published:** 2018-09-13

**Authors:** Peter C Marks, Marc Bigler, Eric B Alsop, Adrien Vigneron, Bart P Lomans, Renato De Paula, Brett Geissler, Nicolas Tsesmetzis

**Affiliations:** 1 Shell International Exploration and Production Inc., Houston, USA; 2 Ecole Supérieure de Biotechnologie Strasbourg, Illkirch-Graffenstaden, France; 3 DOE Joint Genome Institute,Walnut Creek, California, USA; 4 School of Natural and Environmental Sciences, Newcastle University, Newcastle upon Tyne, UK; 5 Shell Global Solutions International B.V., HW Amsterdam, Netherlands; 6 RD & E Microbiology Group, NALCO Champion, Sugar Land, USA

## Abstract

The ever-increasing metagenomic data necessitate appropriate cataloguing in a way that facilitates the comparison and better contextualization of the underlying investigations. To this extent, information associated with the sequencing data as well as the original sample and the environment where it was obtained from is crucial. To date, there are not any publicly available repositories able to capture environmental metadata pertaining to hydrocarbon-rich environments. As such, contextualization and comparative analysis among sequencing datasets derived from these environments is to a certain degree hindered or cannot be fully evaluated. The metagenomics data management system for hydrocarbon resources (MetaHCRs) enables the capturing of marker gene and whole metagenome sequencing data as well as over 300 contextual attributes associated with samples, organisms, environments and geological properties, among others. Moreover, MetaHCR implements the Minimum Information about any Sequence–hydrocarbon resource specification from the Genomic Standards Consortium; it integrates a user-friendly web interface and relational database model, and it enables the generation of complex custom search. MetaHCR has been tested with 36 publicly available metagenomic studies, and its modular architecture can be easily customized for other types of environmental and metagenomics studies.

## Introduction

The amount of data produced from metagenomic studies has dramatically increased with the introduction of massively parallel sequencing technologies (1). Lower sequencing costs and faster processing times have enabled the identification and characterization of microbial assemblages from a number of diverse environments (2). Appraisal of these microbial communities using the 16S rRNA marker gene empowers the taxonomic identification of the community members, whereas whole metagenome sequencing can provide further insights into the prospective biochemical processes, which could be taking place. The comparison of metagenomic datasets from samples originating from different environments can facilitate the identification of genes and pathways that are prevalent (or rare) under certain environmental conditions (3). For example, the presence of elevated numbers of sulfate-reducing bacteria in a petroleum reservoir could indicate favorable sulfate-reducing conditions and the potential for biogenic hydrogen sulfide production, an undesirable process better known as microbial reservoir ‘souring’ (4). Similarly, biofilm growth on either hydrocarbon production or transport infrastructure containing microorganisms known to be implicated in metal corrosion might suggest corrosive conditions. In that manner, it would be possible to distinguish between non-corrosive, corrosive and highly corrosive biofilms and thereby assisting in prediction of likelihood of failure due to microbiologically influenced corrosion (MIC) (4, 5). If left uncontrolled, such microbial processes can lead to adverse environmental and operational consequences.

The ever-increasing metagenomic data necessitate appropriate cataloguing in a way that facilitates the comparison and better contextualization of the underlying investigations. To this extent, information associated with the sequencing data as well as the original sample and the environment where it was obtained from is crucial. Indeed, in some cases, metadata (i.e. the ‘data about the data’) may prove to be as valuable as the sequencing data itself. Data mining of metadata from metagenomic studies has led to significant discoveries through the use of statistical correlations between habitat metadata and metagenomic data (6). The need for accurate, unified and machine-readable metadata has been recommended for standards and semantics (7) in the form of common vocabularies or ontologies, such as the Gene Ontology (8). Specifications, such as the Minimum Information about a Metagenome Sequence, the Minimal Information about a MARKer gene Sequence (MIMARKS) and the Minimum Information about any Sequence (MIxS), have been initiated by the Genomic Standards Consortium (GSC) (9, 10), as a result of the need for metadata standardization or extension of the existing standards (6). More recently, a specification tailored to the metagenomic data derived from hydrocarbon resources (HCRs) (MIxS-HCR) has been proposed (11). The MIxS-HCR package incorporates the core features of the MIxS standard for marker gene (MIMARKS) and metagenomic (MIME) sequences along with HCR customized environmental package. Currently there are efforts to extent this package to cover other Hydrocarbon Occurrences such as ‘Surface or Seabed Geological Structures’ and ‘Anthropogenic Hydrocarbon Occurrences’ (e.g. Oil/Gas Production Systems) (11). Through the MIxS-HCR standard, the comparison and better contextualization of investigations pertaining to the development of hydrocarbon recovery processes and management of microbiological issues in petroleum production systems will be better accomplished.

The need for the storage of metadata related to metagenomic and genomic projects has been addressed by different web platforms or portals and databases (12–17). All these metagenomic-ready platforms are web-based systems accessible publicly or through some user authentication mechanism and—most of them—are hosted at the same institutions as their authors. However, instead of many disjoint databases, there are some obvious advantages in having these systems centralized and publicly available: including the management of the sheer amount of accessible data in them, the ease of data and metadata sharing within the scientific community and the well-supported modern infrastructure. There are disadvantages in having a non-local repository with respect to both data and metadata. First of all, it is impossible to capture environmental metadata that are not currently covered by these online repositories. For example, none of the publicly available repositories are able to capture environmental metadata pertaining to hydrocarbon-rich environments. As such, contextualization and comparative analysis among datasets derived from these environments is to a certain degree hindered or cannot be fully evaluated. Secondly, online repositories can be ‘rigid’ with little room for customization (i.e. use of software tools or parameters other than those already supported). Thirdly, transferring data onto a remote system can be a lengthy process depending on the size and the available bandwidth. Moreover, there may be confidentiality restrictions on the data that could prohibit their transfer and usage outside the institution. This especially concerns private organizations, where such restrictions are a common practice. Furthermore, private organizations may face restrictions on the use of these web platforms for commercial purposes. Users may also wish to use these web resources anonymously without the requirement for registration, which is not an option on some web systems. Finally, the use of a remotely hosted system renders the user dependent on a third-party infrastructure, which is out of one’s control.

Here we describe metagenomics data management system for hydrocarbon resource (MetaHCR), a standalone and open-source software (OSS) that allows users to store, catalogue, share and analyze metadata and sequencing data originating from metagenomics projects related to hydrocarbon environments. MetaHCR consists of a relational database management system for the storage of data and a web application for user interaction with the database. Furthermore, it allows the recording of over 300 different attributes or metadata associated with organisms, the environment, samples, hydrocarbon and water chemistry and geological properties. For this purpose, whenever possible, MetaHCR uses a controlled vocabulary (CV) in association with the MIxS-HCR metadata specification (11) from the GSC. Finally, a user-friendly web front-end enables the non-expert to easily access, interrogate, update, export and analyze the acquired metagenomic data residing in the relational database. MetaHCR is a metagenome data management system suitable for private and public institutions and laboratories who wish to establish their own webserver for the cataloguing and sharing of metagenomic data and metadata related to hydrocarbon-rich environments.

## Program description

### MetaHCR architecture

MetaHCR is a Python application built using the open-source Django web-application framework, and it supports any standard relational database. Django follows the Model-View-Controller architectural pattern. During development, the PostgreSQL database system was used, however, it can be easily replaced by a different database system (e.g. Oracle, SQLServer, etc.) as there is no PostgreSQL-specific code in MetaHCR. All browsing and search requests are translated into standard Structured Query Language (SQL) statements independent of the supporting database. These SQL statements can be viewed using the Django Debug Toolbar (http://django-debug-toolbar.readthedocs.io/en/stable/).

### Deployment of MetaHCR

For the deployment of MetaHCR, any web server supporting Python such as Apache, nginx or Microsoft IIS can be used. MetaHCR can be run on any of the major platforms, like Apple Mac OS X, Microsoft Windows and Linux. MetaHCR can be configured to run in a fully open-source environment, for example by using Linux as the operating system, Apache as the web server and PostgreSQL as the back-end, which is also the recommended configuration. In addition, this configuration makes possible the dissociation of the database from the web application by means of two independent servers.

MetaHCR can be installed through the available installation scripts following the instructions provided in the ‘MetaHCR Installation Guide’ included in the software package. The current MetaHCR version described here is v.1.1. Once installed, a local copy of the MetaHCR can be tested by starting the built-in web server (‘manage.py runserver’), but for security reasons, MetaHCR should be run via a secure web server, such as Apache. Users on the same computer can access MetaHCR by pointing their browsers to the MetaHCR home page: http://127.0.0.1:8000/MetaHCR/home/. The installation guide covers additional use cases, such as deploying MetaHCR on a central production server accessible to every authorized user on the local area network.

The database and web server parts are independent and, as a result, they can be located on different physical servers. Moreover, the databases can be located on more than one physical server. In the simplest case, MetaHCR can be deployed on a local desktop computer, provided that this computer includes a database system, a web server, the appropriate Python and Django support (e.g., mod_wsgi for the Apache web server).

### Database structure

**Figure 1:**
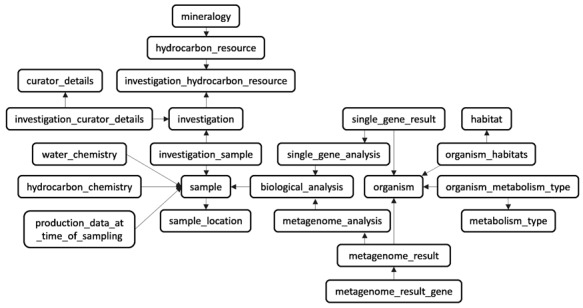
The relational database schema. The database model is divided into 25 tables (23 are shown) with mainly three different types of content (e.g. metadata, static data, and linker tables). The central focus of MetaHCR is the three main tables of ‘investigation’, ‘sample’ and ‘organism’. An investigation can have many samples and a sample can contain many organisms. All the other tables primarily serve as sources for metadata in support of these three tables.

The database schema (Figure 1) uses a traditional model of relational database systems and is structured around a total of 25 distinct tables (Supplementary Table 1). Each table has a specific purpose and most of them support the classification of metadata under categories (e.g. ‘sample’, ‘organism’, ‘hydrocarbon_chemistry’, etc.). Other tables provide more general or static metadata (e.g. ‘country’, ‘contact’, ‘hydrocarbon_resource’) or serve as linkers between two different tables (e.g. ‘investigation_sample’) (Supplementary Table 1).

### Security

The MetaHCR web-interface is protected by a login page requiring a user account and password. By default, without having logged in, an anonymous user can only view the MetaHCR homepage but cannot see, modify, create or interact with the data in any way. Through the security file of the Django web framework, new user accounts can be added to the web application. User account passwords are stored in an encrypted format.

### CV

One of the main goals of MetaHCR is to utilize a CV in the form of ontologies. The use of a CV as semantic metadata can be advantageous in terms of avoiding metadata redundancy, improving data accessibility and facilitating integrated search queries for data retrieval. To achieve this goal, the database schema of MetaHCR implements a table named ‘attribute’ (not shown in Figure 1), which categorizes the CV or metadata as attributes and can contain one or many unique values. In addition, it is possible for each metadata value to have further descriptors, such as source type and source details and even a boolean flag in case this metadata value is deprecated. The metadata values from the ‘attribute’ table can then be referred to by other tables, using their unique identifier integer through a one-to-many relationship. There are over 849 different metadata attributes in all of the combined tables.

During installation of MetaHCR, it is possible to pre-populate the ‘attribute’ table with a core set of metadata values for the projects, sequencing, organisms, habitats, samples and geology categories. The values of this core set of metadata originate mostly from literature review or biological and biomedical ontologies (18). For those originating from ontologies, the Open Biological and Biomedical portal (abbreviated as BioPortal) (19) has been employed for finding the most appropriate set of metadata values for specific attributes. Accordingly, sequence ontology (20) was used to describe the quality of sequencing, and Habitat-Lite (21), an easy-to-use subset of terms from the Environment Ontology (22), was used to characterize an organism’s habitat.

### Web interface

**Figure 2:**
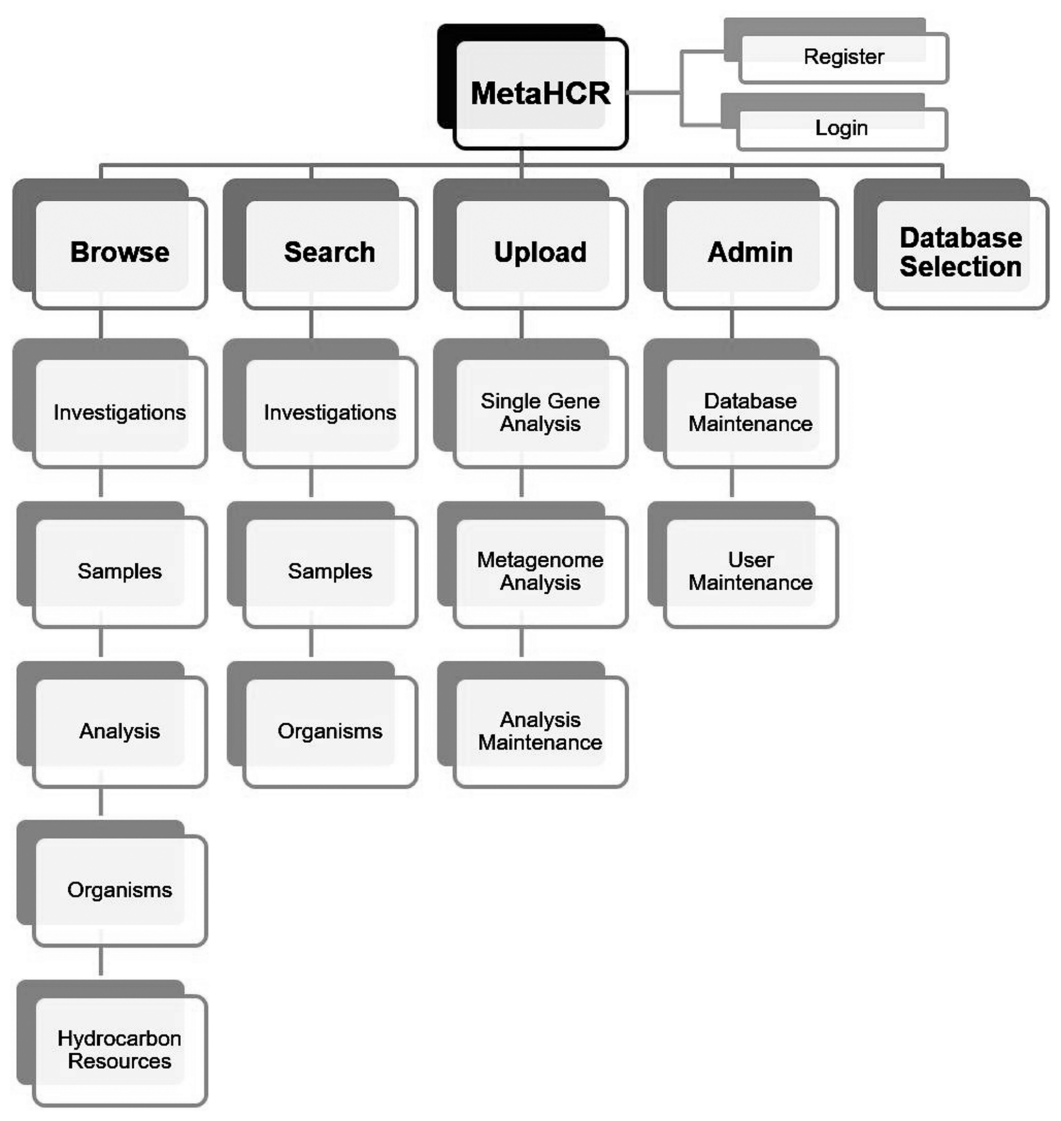
MetaHCR web interface components map. The MetaHCR web application is segmented into five different components (bold) providing one or more specific and component-related functionality. All functionalities are at any time available in the web application through a navigation menu in addition to the register and login actions.

MetaHCR is accessible through a user-friendly web front-end interface segmented into various components depending on the desired functionality. These components are available from any part of the web application, through a simple navigation menu. Consequently, the menu gives immediate access to the following components: browse, search, upload, administrate and change databases with their respective sub-components or actions (Figure 2). A more detailed description of the web page interface appears in subsequent sections.

Additionally, a ‘register’ link allows a new user to create an account in MetaHCR and a ‘login’ link lets a user who is in possession of a valid account to login. An icon followed by the name of the database will remind the user which database is connected to at any time. Furthermore, an informational box will provide specific success or failure messages on any kind of user action, as well as general help and hints regarding the component in use.

### Browse component

**Figure 3:**
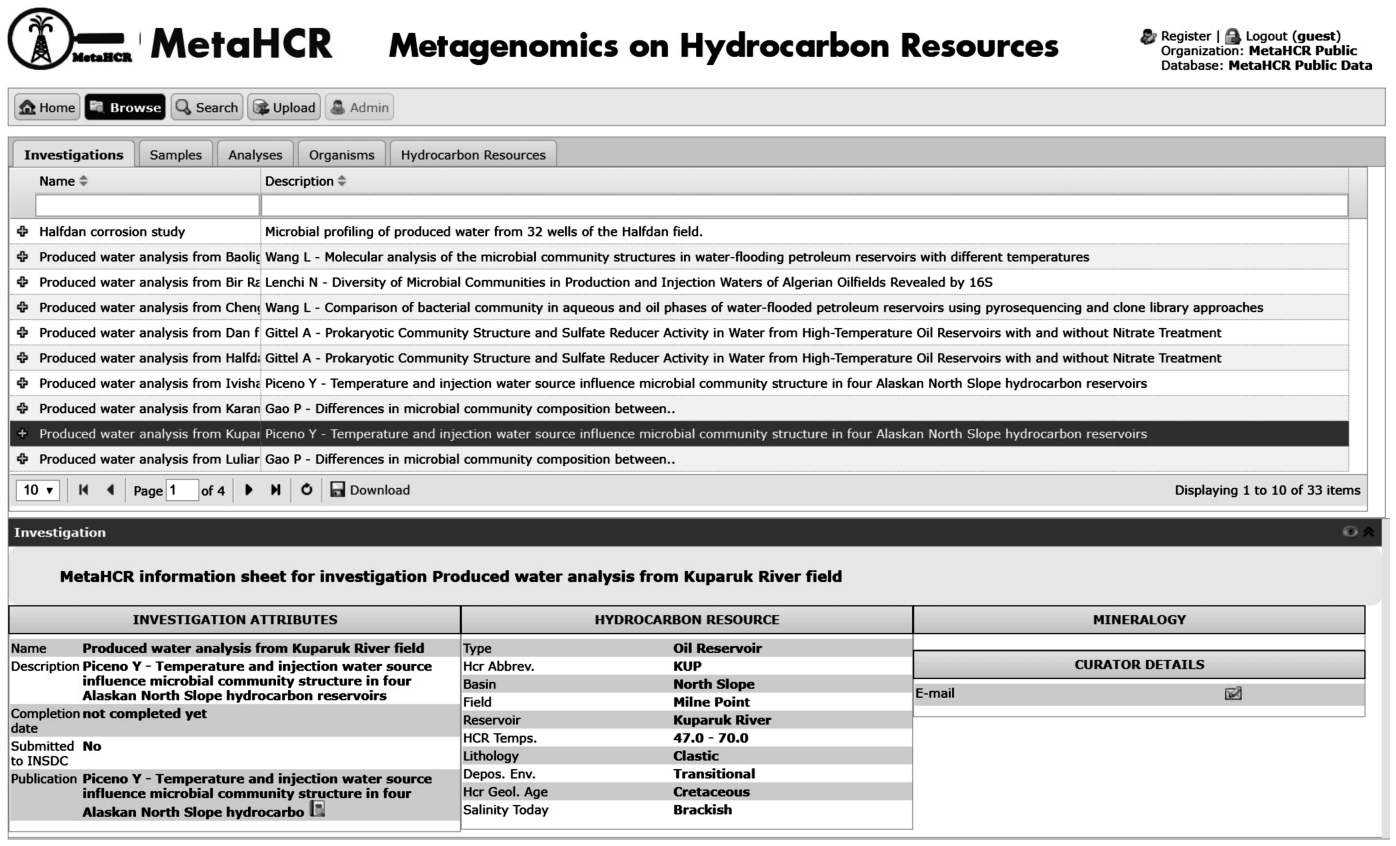
Screenshot of an Investigation infosheet. Display of information related to the investigation such as type of samples analyzed, reference to any associated publications (linked to PubMed), description of the HCR (e.g. temperature, geology, salinity, lithology, etc.) and other relevant information (when available).

In a nutshell, the browse component provides the user with the ability to sort, make simple queries and export the data stored in the most important tables of the database. These are the ‘investigation’, ‘sample’, ‘analyses’, ‘organism’ and ‘HCR’ tables. Clicking on the tab associated with each table displays its contents in a table-formatted list. It is possible to sort data by any column in ascending or descending order. Typing values into the search field located at the top of each column starts a real-time search of the data associated with that tables column. The columns that have a restricted set of values (i.e. CVs) feature a drop-down list from which to choose the search value. Clicking on any row brings up an ‘infosheet’ i.e. a formatted display that is associated with the particular entity (Figure 3).

**Figure 4:**
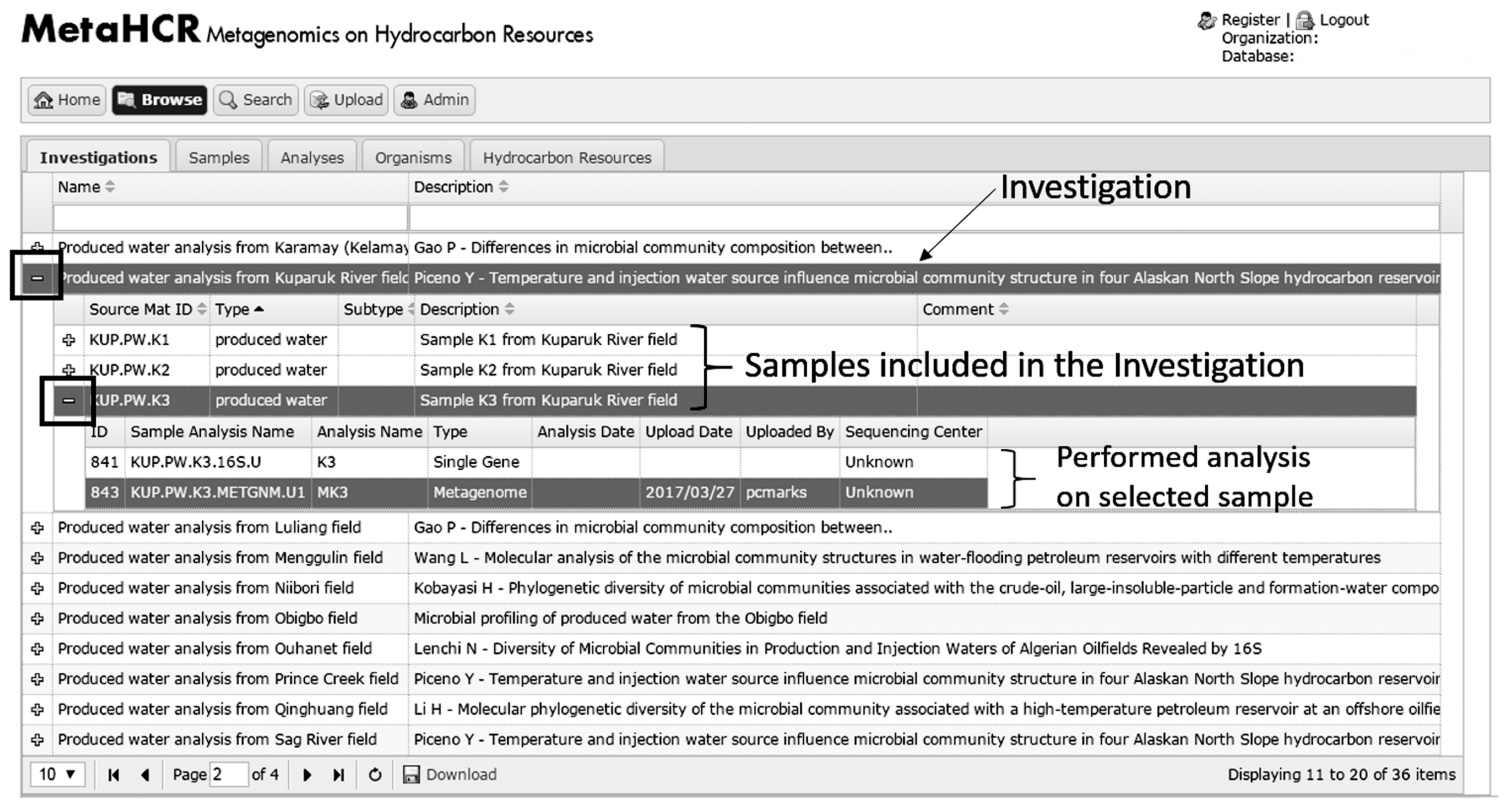
Screenshot of the expandable sub-table data association. Samples associated with a particular investigation can be visualized in the form of a sub-table by clicking on the plus (+) sign. In turn, available analyses for a particular sample can be viewed by expanding the associated sub-table using the plus sign right next to the sample name.

Another feature available in the browse component is the ability to explore samples and analyses that are associated with an investigation. While browsing the ‘investigation’ table, if a particular investigation has one or more samples associated with it, a plus-sign (+) appears in the left-most column. Clicking this plus-sign will open up a sub-table listing all the sample(s) associated with this particular investigation (Figure 4). If the Sample has analyses, then a plus-sign will appear next to the sample row. Clicking on the plus-sign will list all the different analyses performed on this sample. A similar functionality is also available when browsing the ‘sample’ table. The corresponding infosheets are also available in these nested displays.

### Search component

**Figure 5:**
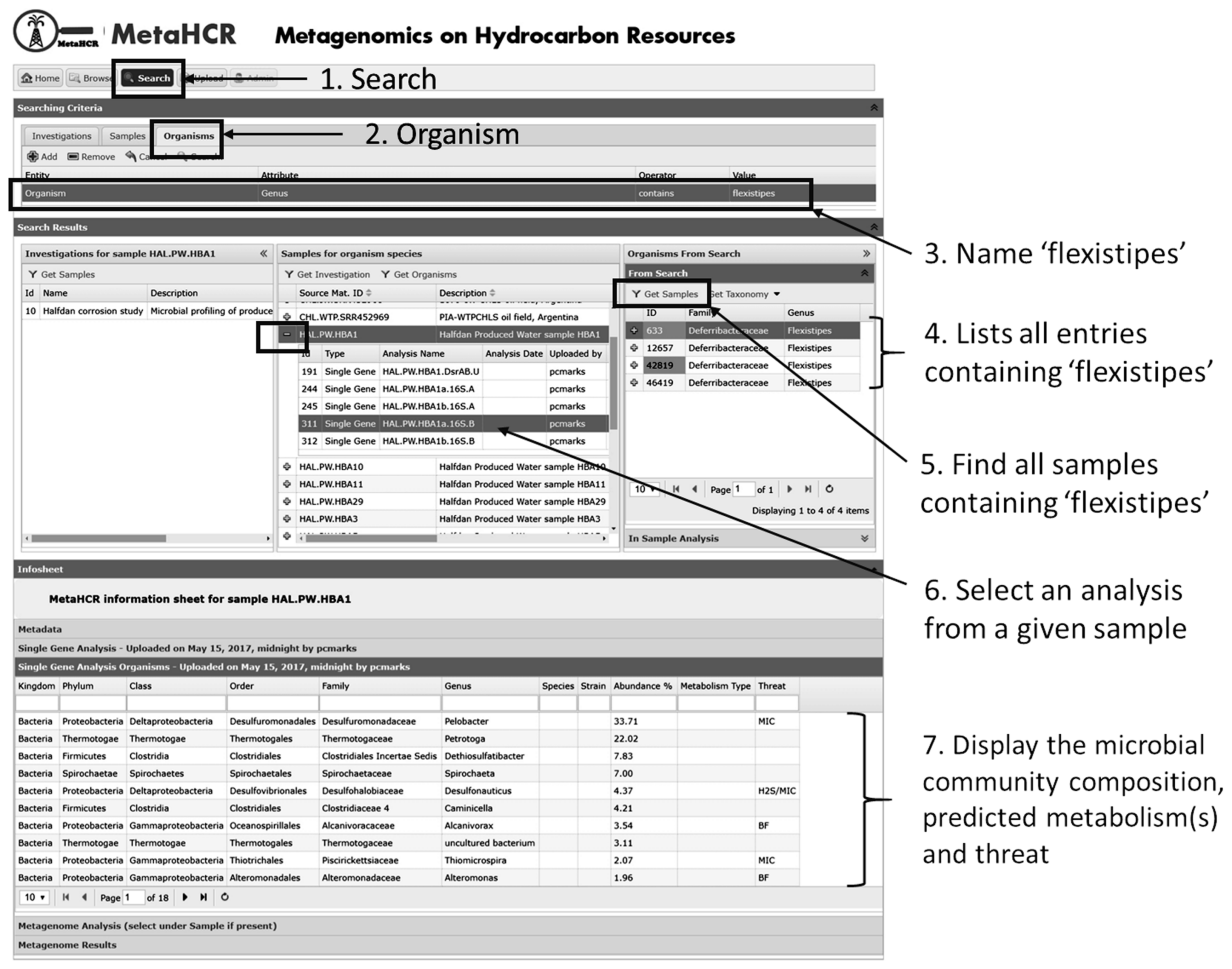
Example of search functionality. The search functionality provides an intuitive way to search for organisms and contextual data. In this example, the organism *Flexistipes* was searched in an effort to find any samples associated with this organism (steps 1–5). In addition, all organisms identified in a given sample can be listed along with their predicted metabolisms and threat they may pose to the production system (steps 6 & 7).

The search component enables the searching of investigations, samples and organisms and, importantly, the relationships between these entities. For example, given an investigation, the user can find all the samples that are associated with that investigation. Similarly, given a sample, the user can find all the organisms that were identified in it. Finally, given an organism, it is possible to find all samples that this organism has been found in which can, in turn, point to the investigation associated with the sample (Figure 5).

The search functionality enables the user to create a customized search query using metadata attributes and combining them through the use of the AND and OR boolean logic operators. For example, it is possible to find samples given certain hydrocarbon chemistry values (or ranges). The search component recognizes three different types of attributes: numeric, free text and CV. Once the criteria are specified, clicking on the ‘Search!’ button will begin the search. The results are shown in the ‘Search Results’ area of the display.

The search results area is divided into three sections corresponding to investigations, samples and organism. The values in each section can be used to follow the relationships between ‘Investigations’, ‘Samples’ and ‘Organisms’ by clicking on the ‘Get Investigation’, ‘Get Samples’ and ‘Get Organisms’ buttons (Figure 5).

### Upload component and data storage

The upload component of MetaHCR facilitates the uploading of single gene and metagenome analyses. Moreover, MetaHCR can also handle the storage of large amounts of data associated with single gene and metagenome analyses in a central location. The location of this storage is configurable by the administrator. As an example, MetaHCR comes configured with the ability to store analyses source files and their upload log files in Amazon’s AWS S3 storage—a cloud-based and secure storage facility. Alternatively, MetaHCR can be configured to store these files on local file systems.

### Single gene and metagenomic data cataloguing

Taxonomic summary tables produced by amplicon processing pipelines like QIIME and mothur (23, 24) are imported in MetaHCR and can be visualized in the organism panel as well as in the corresponding ‘single gene results’ info-sheet. In addition to the relative abundance of each of the identified taxa, information on the predicted predominant metabolism(s) and the potential threat this taxon may pose to the hydrocarbon production system are also displayed if known. Prediction of microbial metabolism is based on the presence of key metabolic enzymes (e.g. ‘nar’, ‘dsr’, ‘mcr’, etc.) on the closely related genomes in IMG or on references in the public literature. Where more than one metabolic enzymes are detected, all relevant metabolisms are displayed in the ‘Metabolism Type’ field. Depending on the type of metabolism detected on a taxon, a potential damaging role is assigned to it. For example, if a member of the genus *Desulfovibrio* appears in the taxonomic summary, sulfate reduction will be assigned as its predicted metabolism and souring (i.e. biogenic production of hydrogen sulfide) would be its potential damaging role (i.e. threat) to the hydrocarbon production system. Metabolic predictions and threat assignments, when known, are made to all taxa residing in MetaHCR.

**Figure 6:**
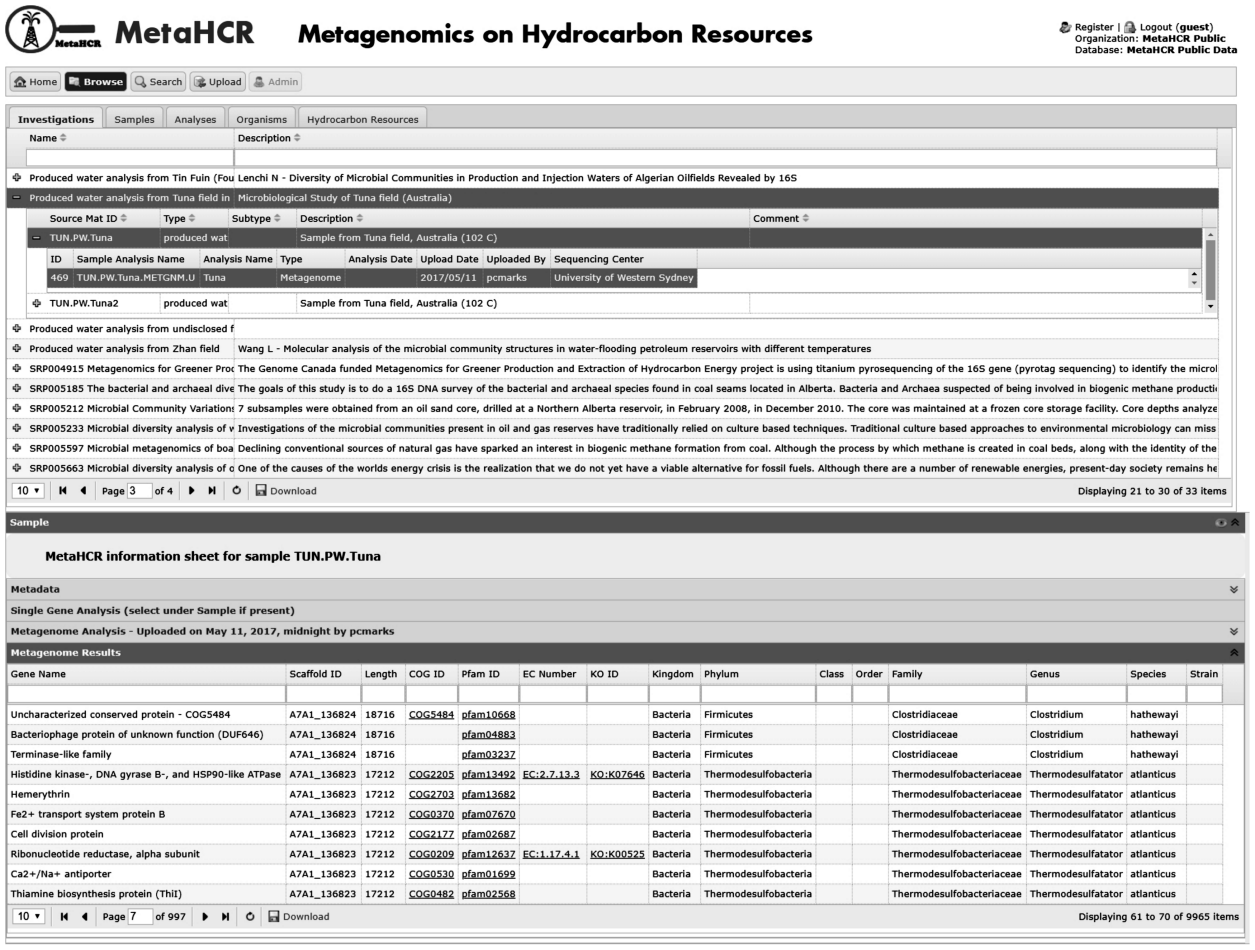
Screenshot of a metagenome analysis infosheet listing all genes identified by the IMG/M annotation pipeline along with associated information (e.g. taxonomy, functional domains, enzyme commission identifiers, etc.). Any of the listed fields can be searched in real time using the provided search fields shown underneath field names.

Metagenomic data captured in MetaHCR rely on IMG’s annotation pipeline. Metagenomic information related to predicted genomic features (e.g. gene name, location, orientation, length, scaffold name, etc.), functional assignments (e.g. COG, Pfam, EC number, KO, etc.) and taxonomic affiliations are imported into MetaHCR and exhibited under sample’s ‘metagenome results’ tab (Figure 6). Hyperlinks to external resources are also provided where possible (COG, Pfam, EC number and KO). All metagenomic fields are searchable in real-time using the search field provided at the top of each table column. For example, a user can search a metagenomic dataset for potential nitrate-reducing microorganisms by either typing ‘nitrate reductase’ in the ‘gene name’ field or by searching for any corresponding identifiers for this gene in the appropriate column (e.g. KO: K00370, EC:1.7.5.1, COG: COG5013, etc.).

### Intended use and benefits

The MetaHCR data management system aims to be a platform for the cataloguing, data storage, sharing and searching for microorganisms from environmental samples that have been recovered from HCRs and that are associated with hydrocarbon degradation, biogenic H_2_S production, MIC and methane emissions. More broadly speaking, MetaHCR is a platform for a census of metagenomic data including all the relevant metadata for organisms (and their predicted metabolic functions) that are directly or indirectly related to HCRs. When a database contains a significant amount of valuable data and metadata, it is expected to be used as a knowledgebase in order to gain a better understanding of biological and biochemical processes taking place at these sites. Furthermore, it is intended to be utilized as a central repository for DNA sequences originating from metagenomic sequencing and thus to avoid using local desktops for file storage. In the long-term, it may also phase out the use of spreadsheets on desktop or laboratory computers for the recording of metadata information, such as those related to a sample, sequencing run or organism. Therefore, MetaHCR aims at being a centralized management system for recording biological and associated metadata while it facilitates easy data sharing among collaborators or third-party institutions, when needed. MetaHCR is built on a clear and well-segmented data model and offers a user-friendly and simple web interface, which makes it accessible even to the non-expert. On the administration side, since the data are centralized, it greatly facilitates the support, maintenance and creation of data backups by simply using the database’s built-in backup tools. In addition, migration of data to another server or similar platform is made simple. Currently, data exchange between different databases can be achieved by either using the database’s ‘dump’ and ‘restore’ functions or by CSV (comma-separated values) files. Data essential for the proper function and sharing between MetaHCR instances include those in the Attributes table as they comprise the ontological terms (i.e. CV) used in many of table fields in the MetaHCR database. MetaHCR is a standalone data management system and therefore can be deployed locally within a private organization for additional flexibility and security. Alternatively, MetaHCR can be deployed as a distributed database with the maintenance and updating being provided by a central authority. In this case, the implementation of the notion ‘Organization’ who provides access to only a set of authorized MetaHCR users can be particularly useful. Using the ‘Organization’ notion, a service company that offers its services to multiple private organizations can restrict the data that are shared with each of its customer organizations.

Serving the role of an application programming interface (API), many of MetHCR’s web application’s requests for data are implemented as HTTP requests (with suitable parameters); the server responses are formatted as JSON. Examples of these requests include listing all Investigations, Samples, Analyses and Organisms or listing all Samples associated with an Investigation, or listing all analyses associated with a Sample or exporting Single Gene and Metagenome Analyses. Most of the above requests (and others) can take one or more ‘filters’ (i.e. restrict responses to record field values that meet certain criteria).

Finally, as MetaHCR has been built on OSS and is distributed as an OSS, it allows anyone with programming skills to modify, extend, improve or just examine its source code at any time. Moreover, by being OSS, MetaHCR alleviates the requirement for financial investment on such software.

### Performance

The Django web development framework, which is used by MetaHCR, is stable, robust and highly responsive. Moreover, it has been designed to support web applications that are secure and scalable. Horizontal scalability for accessing MetaHCR can be realized with the use of multi-threaded web servers such as Apache and Internet Information Services. For databases, such as PostgreSQL, the replication of database servers can be employed.

User interaction has been enhanced by using JavaScript on the client side. More specifically, MetaHCR has been built using the AJAX methodology, which makes a web application more collaborative, rapid and dynamic. Further, it reduces the server workload and increases the application’s responsiveness.

On the database side, the schema has been designed to allow for more efficient queries by splitting data into smaller and logically grouped tables. Database indexes are also used with the relevant table columns in order to allow queries to retrieve data significantly faster especially when the database contains many entries.

### Comparison with similar software

To our knowledge, this is the first publicly available software**—**commercial or open source**—**capable of storing, cataloguing and sharing all the relevant metagenomic data and associated metadata from hydrocarbon-rich environments.

Although no similar standalone software is currently available, there are several metagenomic-related online web platforms available on the internet. However, none of them is targeted to environments pertaining to HCRs. The majority of these web portals are intended to be either universal (i.e. GOLD, IMG/M, MG-RAST) (13, 15, 16) or tailored to a specific environment like megx.net (12), which focuses on marine metagenomics. Additionally, IMG/M and MG-RAST offer supplementary services, such as shared access to their high-performance computing infrastructure for automatic annotation. On the contrary, MetaHCR does not provide any kind of automated metagenome annotation or community analysis, but it is rather designed to be used in collaboration with other metagenomic tools for automated sequence analysis, such as QIIME for marker gene surveys or IMG/M for metagenomic studies.

Owing to open and modular architecture of MetaHCR, components may be added or interchanged. Therefore, additional metadata categories for other types of environmental and metagenomics studies, such as marine microbiology, can readily be added without the need to reprogram the application.

### Future improvements

One of the improvements from which MetaHCR would benefit most is the incorporation of data visualization tools such as Krona plots (25) for a more elegant representation of taxonomic or functional data compared to the current tabular forms. Other improvements pertain to the incorporation of corrosion attributes making it suitable for MIC related samples, enhancements on the way large sets of data are imported in MetaHCR as well as interconnecting multiple MetaHCR instances for synchronous data sharing. The latter would be particularly useful for sharing data between two or more institutions.

## Conclusions

The software presented here may be helpful for various tasks related to the management and investigation of complex metagenomics data and metadata for hydrocarbon-rich environments. MetaHCR greatly facilitates the recording of relevant metadata, as well as the standardization of data, using CV whenever possible. Export of metadata or search results is facilitated by downloading these data in a CSV-formatted file readable by any spreadsheet software. MetaHCR integrates a user-friendly and modern front-end web interface to interact with the database back-end, which makes the application accessible to the non-expert. Moreover, it has been exclusively built using OSS and cutting-edge technologies, such as the Django web application framework. Equally important, the software has been extensively tested with 36 metagenomic investigations pertaining to HCRs retrieved from public raw data repositories. This is currently the first public release of the fully functional and expandable MetaHCR software for metagenomic studies.

## Supplementary Material

Supplementary Data

## Notes

**Database URL**: https://github.com/metahcr
