# Supplementary material for: MetaHCR: a web-enabled metagenome data management system for hydrocarbon resources
Source: Database (Oxford). 2018 Sep 13;2018:bay087. doi: 10.1093/database/bay087 (PMC6146120; doi:10.1093/database/bay087)
Supplement: Supplementary Data [file bay087_supp.docx]

| **Name** | **Type** | **Description** |
| --- | --- | --- |
| *attribute* | - | Storage for unique metadata values and controlled vocabulary |
| *biological_analysis* | - | Super class for single_gene_analysis and metagenome_analysis |
| *country* | Static | ISO 3166 formatted list of country codes and names |
| *curator_details* | Static | Contact details of a person associated to an investigation. |
| *habitat* | - | Controlled vocabulary of organism habitats. |
| *hydrocarbon_chemistry* | Metadata | Metadata for the chemistry of hydrocarbon. |
| *hydrocarbon_resource* | Static | Metadata of worldwide hydrocarbon resources. |
| *investigation* | Metadata | Placeholder and main table for metagenomic analyses. |
| *investigation_curator_details* | Linker | Link between an investigation and its curator(s). |
| *investigation_hydrocarbon_resource* | Linker | Link between an investigation and its hydrocarbon resource. |
| *investigation_sample* | Linker | Link between an investigation and its sample(s). |
| *metabolism_type* | - | Controlled vocabulary of organism metabolism types. |
| *metadata_info* | - | Descriptive metadata. |
| *metagenome_analysis* | Metadata | Metagenomic analysis metadata and results |
| *metagenome_result* | Metadata | Metagenomic analysis results (organisms). |
| *metagenome_result_gene* | Metadata | Metagenomic analysis gene results. |
| *mineralogy* | Metadata | Percentage of main minerals present and associated to a geology entity. |
| *organism* | Metadata | Metadata associated with an organism. |
| *organism_habitats* | Linker | Link between an organism and its habitat(s). |
| *organism_metabolism_type* | Linker | Link between an organism and its metabolism type(s). |
| *production_data_at_time_of_sampling* | Metadata | Production metadata related to a sample. |
| *sample* | Metadata | Metadata associated with a sample. |
| *sample_location* | Metadata | Metadata related to a samples location. |
| *single_gene_analysis* | Metadata | Single gene analysis metadata |
| *single_gene_result* | Metadata | Single gene analysis results (organisms). |

**Supplementary Table 1**. **Description of MetaHCR’s database tables including the type of data being stored.**
